# Outcomes in Patients With Lung Adenocarcinoma With Transformation to Small Cell Lung Cancer After EGFR Tyrosine Kinase Inhibitors Resistance: A Systematic Review and Pooled Analysis

**DOI:** 10.3389/fonc.2021.766148

**Published:** 2022-01-28

**Authors:** Jinhe Xu, Lihuan Xu, Baoshan Wang, Wencui Kong, Ying Chen, Zongyang Yu

**Affiliations:** ^1^ Fu Zong Clinical Medicine, Fujian Medical University, Fuzhou, China; ^2^ Department of Gastroenterology, Dongfang Hospital of Xiamen University, Fuzhou General Hospital of Fujian Medical University, The 900th Hospital of the Joint Logistic Support Force, PLA, Fuzhou, China; ^3^ Fuzhou General Hospital of Fujian Medical University, Dongfang Hospital of Xiamen University, Department of Respiratory and Critical Care Medicine, The 900th Hospital of the Joint Logistic Support Force, PLA, Fuzhou, China

**Keywords:** lung adenocarcinoma, epidermal growth factor receptor (EGFR), transformation, small-cell lung cancer (SCLC), systematic review

## Abstract

**Background:**

Lung adenocarcinoma can transform into small-cell lung cancer (SCLC) when resistance to tyrosine kinase inhibitors (TKIs) develops. Approximately 3% to 10% of epidermal growth factor receptor (EGFR)-mutant non-small cell lung cancer (NSCLC) could transform to SCLC. This phenomenon has been described in several case reports and small patient series. However, the characteristics and treatment outcomes of this population have not been comprehensively reported, and their clinical course is poorly characterized.

**Methods:**

We performed a systematic review of the published literature to summarize the clinical and pathological features and prognosis of the reported cases and analyzed the demographics, disease features, and outcomes.

**Results:**

A total of 72 patients (50 females and 22 males) initially diagnosed with lung adenocarcinoma were included. EGFR mutations included 19-deletion (75%), L858R (22%), and G719X (3%). All patients received EGFR-TKIs before SCLC transformation. The median time from diagnosis to transformation was 20.5 months (95% CI, 15.45 to 26.55 months). Of the 67 patients with post-translational gene test results, 58 maintained their EGFR mutation, and only 1 of 18 with prior T790M positivity retained T790M mutation. After the pathological transformation, both conventional chemotherapy regimen and chemotherapy combined targeted therapy yielded high response rates. The disease control rate of first-line therapy after transformation was 76%, while the objective response rate was 48%. The median overall survival (OS) since diagnosis was 27 months (95% CI, 22.90 to 31.10 months), whereas median OS since SCLC transformation was 8.5 months (95% CI, 5.50 to 11.60 months).

**Conclusion:**

The prognosis of transformed SCLC is worse than primary SCLC. The response rate to conventional chemotherapy was high. However, the progression-free survival and OS after transformation were short and the prognosis was poor with first-line therapies. New therapies are needed in the management of transformed SCLC.

## Introduction

Lung cancer (LC) is the leading cause of cancer-related death. The 2020 Globocan project (1) reported an estimated 2.2 million new cases of LC globally. In the past decades, two broad histological subtypes of LC have been recognized, non-small cell lung cancer (NSCLC) and small-cell lung cancer (SCLC). These two subtypes are commonly reckoned as different diseases due to their distinct biology and genomic characteristics. NSCLC represents about 85% of all cases, arising from the respiratory epithelium, and is further divided into adenocarcinoma and squamous-cell carcinoma. SCLC, accounting for the remaining 15% of cases, is a highly aggressive tumor of neuroendocrine origin, which is characterized by rapid disease progression and early development of metastases (2–4). However, the concept of small cell transformation (SCT) links them together. Histological transformation of epidermal growth factor receptor (EGFR) mutant lung adenocarcinoma (LADC) into SCLC was first reported in 2006 (5).

With the discovery of a series of LC-driven genes, many studies in China and worldwide have shown that targeted therapeutic drugs greatly improve and prolong the prognosis and survival of patients with NSCLC carrying corresponding driven genes (6–10). Forty percent to 50% of patients with LADC have EGFR-sensitive mutations (3). The only two common activating and sensitizing EGFR mutations included in prospective clinical trials are 19-deletion (19-del) and exon 21-L858R (21-L858R) point mutations. Ten percent to 20% of patients with NSCLC harbor uncommon EGFR mutations that have variable sensitivity to different EGFR TKIs. However, progression of the disease is inevitable after a median time of approximately 9.0–12.0 months (11, 12). The main resistance mechanism of EGFR-TKIs is the emergence of secondary EGFR-T790M mutations. Among the first-/second-generation EGFR-TKIs-resistant patients, 50% of patients have secondary EGFR-T790M mutations (13–15). Repeated biopsy cohort studies have shown that approximately 3%–10% of acquired EGFR-TKIs resistance is related to histological transformation to SCLC (16, 17).

The mechanism of SCLC transformation remains unclear. Several studies have shown that alveolar type II cells may be common precursors of both LADC and SCLC (4, 18, 19). LADC arising from alveolar type II cells and harboring EGFR mutations might trans-differentiate to SCLC under the selective pressure of TKI therapy. Genomic sequencing of tumor samples from repeated biopsies of LADC progressing on TKIs revealed that most SCLC retained the same EGFR mutation type of the LADC counterpart (20). Only a few case reports and small series have been published due to the rarity of this phenomenon. In 2017, Roca et al. (21) reviewed the literature of reported cases for SCLC transformation. However, the study did not strictly select the criteria for inclusion, and the number of transformation cases was only 39.

In this study, we once again reviewed the literature of all reported cases of SCLC diagnosed in patients treated with TKIs for EGFR-mutated LADC. Excluded cases initially mixed with SCLC components and ineffective TKIs treatment. The aim was to obtain explorative information on the clinical and pathological characteristics and the prognosis of the identified patients with a transformed SCLC phenotype.

## Patients and Methods

### Search Strategy

**Figure 1:**
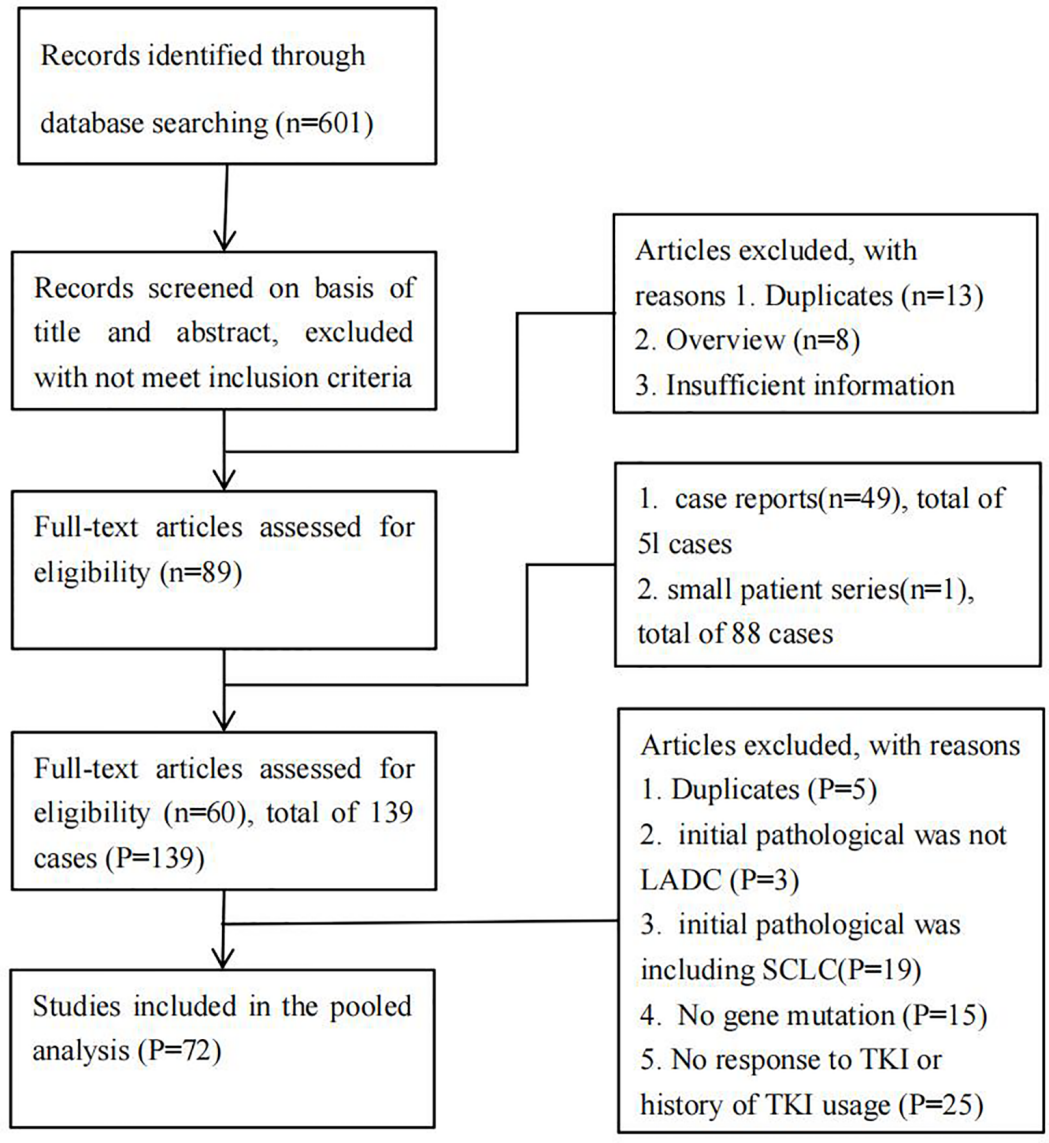
PRISMA flowchart.

JX and LX respectively systematically searched the literature using PubMed/Medline (US National Library of Medicine National Institutes of Health) and EMBASE (2006 to present) with the following keywords: “transformation from NSCLC to SCLC”, “NSCLC transformation in SCLC”, “resistance to TKIs”, and “TKIs treatment”. Search results were limited to human studies in English. A manual review of reference lists in relevant publications was also carried out to identify additional articles. Conversely, abstracts from scientific meetings were not included. For duplicated publications, we selected the most recent version. The PRISMA flow diagram showing the selection process for this systematic review is depicted in **Figure 1**.

### Inclusion and Exclusion Criteria

Inclusion criteria: (1) patients with LADC diagnosed with EGFR mutation; (2) a prior history of using EGFR-TKIs treatment before the transformation of LADC, including the first/second/third generation; (3) EGFR-TKIs was used for at least 3 months and the efficacy evaluation was at least stable disease (SD); and (4) SCLC should be diagnosed in high-quality tumor biopsies or well-preserved cytological samples according to 2015 WHO classification (22).

### Statistical Analysis

Data that were collected included demographic information, tumor histology, molecular pathology, clinical treatments, and outcomes of all published patients that were extracted from the full-length articles by JX and LX. Data were included in a specific database and analyzed as a case series. We defined the time to SCLC transformation (ttSCLC) as the time from the initial pathological diagnosis of LADC to the additional biopsy revealing the metachronous SCLC phenotype. We defined the T-ttSCLC as the time from the initial TKIs usage to the additional biopsy revealing the metachronous SCLC phenotype. Disease control rate (DCR) was defined as the percentage of patients with complete response or partial response or stable disease.

Survival curves were illustrated using the Kaplan–Meier method and compared with the log-rank test. Single-factor exploratory analyses were performed using Cox proportional hazards regression model to test the prognostic value of patient and tumor characteristics [hazard ratios (HR) and 95% confidence intervals (CIs)] for overall survival (OS). *p*-values <0.05 (two-sided) were considered statistically significant. Kaplan–Meier survival curve chart was used to assess the validity of the proportional hazard assumptions. The primary end point of first-line treatment after transfer was objective response rate (ORR) by independent radiology assessment. ORR was defined as the percentage of patients with at least one visit response of complete response or partial response confirmed. All statistical analyses were performed using Statistica software, version 8 (StatSoft Inc., Tulsa, OK) and SPSS, version 25.0 (SPSS, Chicago, IL). GraphPad Prism version 8.0 is used for graphing.

## Results

Our search strategy identified a total of 601 articles (**Figure 1**). Among these, 42 articles were relevant reports of patients with the histopathological transformation from LADC to SCLC after TKIs therapy (4, 16, 18, 23–61). Of these 42 articles, 32 were case reports (4, 23–53), and 10 were small patient series (16, 18, 54–61). A total of 72 patients with transformed SCLC from a prior LADC were identified (**Appendix Table**).

### Pre-Transformation Characteristics

#### Patient Characteristics

**Table 1** summarized the characteristics of the 72 identified patients. The median age of patients was 56.5 years (range 31 to 76 years). Twenty two were males (30.6%) and fifty were females (69.4%); 70.8% of the patients were Asian and 29.2% were white. The smoking status, available in 64 patients, was positive in nineteen patients (29.7%), including former smokers. Forty-five patients (70.3%) were never smokers; 95% of the cases were diagnosed with advanced LADC (TNM stage III or IV). All cases had positive EGFR mutation. The EGFR mutation profile was as follows: exon 19-deletion in 54 patients (75%), exon 21-L858R in 16 patients (22.2%), and exon 18-G719X in 2 patients (2.8%). In one case, the EGFR status was wild type at diagnosis but became mutated on exon 19 at disease progression, suggesting a false-negative result at first diagnosis.

**Table 1:** Clinical characteristics of patients with advanced EGFR-mutated LADC transforming to SCLC.

Characteristics	Total (*n* = 72)	ttSCLC (95% CI)	T-ttSCLC (95% CI)
Age			
Median (range)	56.5 years (31–76 years)	20.5 m (3–75 m)	18.5 m (3–64 m)
Gender (%)			
Male	22 (30.6%)	14.0m (14.58–26.07 m)	14.0 m (13.48–23.72 m)
Female	50 (69.4%)	21.5 m (22.00–30.88 m)	19.5 m (18.91–26.28 m)
Race (%)			
Asian	21 (70.8%)	22.0 m (18.44–31.8 m)	19.0 m (16.47–28.10 m)
White	51 (29.2%)	20.0 m (20.03–28.64 m)	17.0 m (17.45–24.56 m)
Smoking (%)	*n* = 64		
Never smoking	19 (29.7%)	20.0 m (21.12–31.02 m)	18.0 m (17.65–25.55 m)
Ever smoking	45 (70.3%)	20 m (14.68–23.63 m)	20 m (13.75–2.51 m)
TNM stage			
II	4 (5.5%)	44.5 m (10.20–72.80 m)	18.0 m (5.62–37.38 m)
III	7 (9.7%)	29.0 m (18.05–38.67 m)	23.0 m (15.36–26.72 m)
IV	61 (84.7%)	20.0 m (19.23–27.71 m)	18.0 m (18.17–25.05 m)
Founder EGFR mutation (%)			
19-del	54 (75.0%)	21.0 m (20.78–29.29 m)	19.0 m (18.13–25.11 m)
21-L858R	16 (22.2%)	20.0 m (16.93–31.20 m)	20.0 m (14.59–28.35 m)
18**-**G719X	2 (2.8%)	14.0 m	14.0 m
First line TKIs type using (%)			
Gefitinib	33 (45.8%)	20.0 m (19.38–30.57 m)	17.0 m (16.41–26.33)
Erlotinib	24 (33.3%)	22.0 m (19.60–32.24 m)	20.0 m (17.56–26.19)
Afatinib	9 (12.5%)	19.0 m (10.17–34.94 m)	18.0 m (9.12–32.88)
Icotinib	4 (5.5%)	16.0 m	16.0 m
Osimertinib	2 (2.7%)	18.0 m	18.0 m

m, months; TKIs, Tyrosine kinase inhibitors; 19del, EGFR exon 19-deletion; 21 L858R, EGFR exon 21-L858R; 18-G719X, EGFR exon 18-G719X; TNM stage, the stage at the time of diagnosis; ttSCLC, the time from the initial pathological diagnosis of LADC to the additional biopsy revealing the metachronous SCLC phenotype; T-ttSCLC, the time from the initial TKIs usage to the additional biopsy revealing the metachronous SCLC phenotype.

Among the 72 patients, 64 patients (88.8%) received TKIs as first-line therapy, 7 patients (9.7%) as second-line therapy, and 1 patient (1.3%) as third-line therapy. Sixty-one patients (84.7%) received 1st-generation TKIs (Erlotinib, Gefitinib, Icontinib), nine patients (12.5%) received 2nd-generation TKI (Afatinib), while only 2 patients (2.7%) were treated with a 3rd-generation TKI (Osimertinib).

#### Predictors of SCLC Transformation

**Figure 2:**
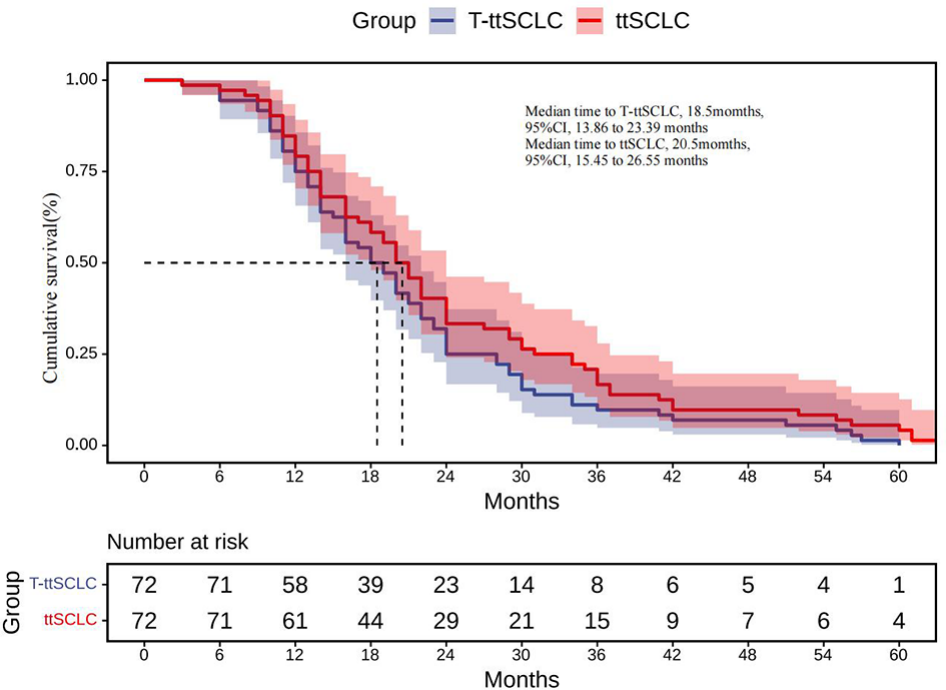
ttSCLC, Kaplan–Meier estimates of time from initial diagnosis of LADC to development of SCLC phenotype; T-ttSCLC, Kaplan–Meier estimates of time from start of TKI treatment to development of SCLC phenotype. LADC, Lung adenocarcinoma; SCLC, Small cell lung cancer; TKI, Tyrosine kinase inhibitor.

**Figure 3:**
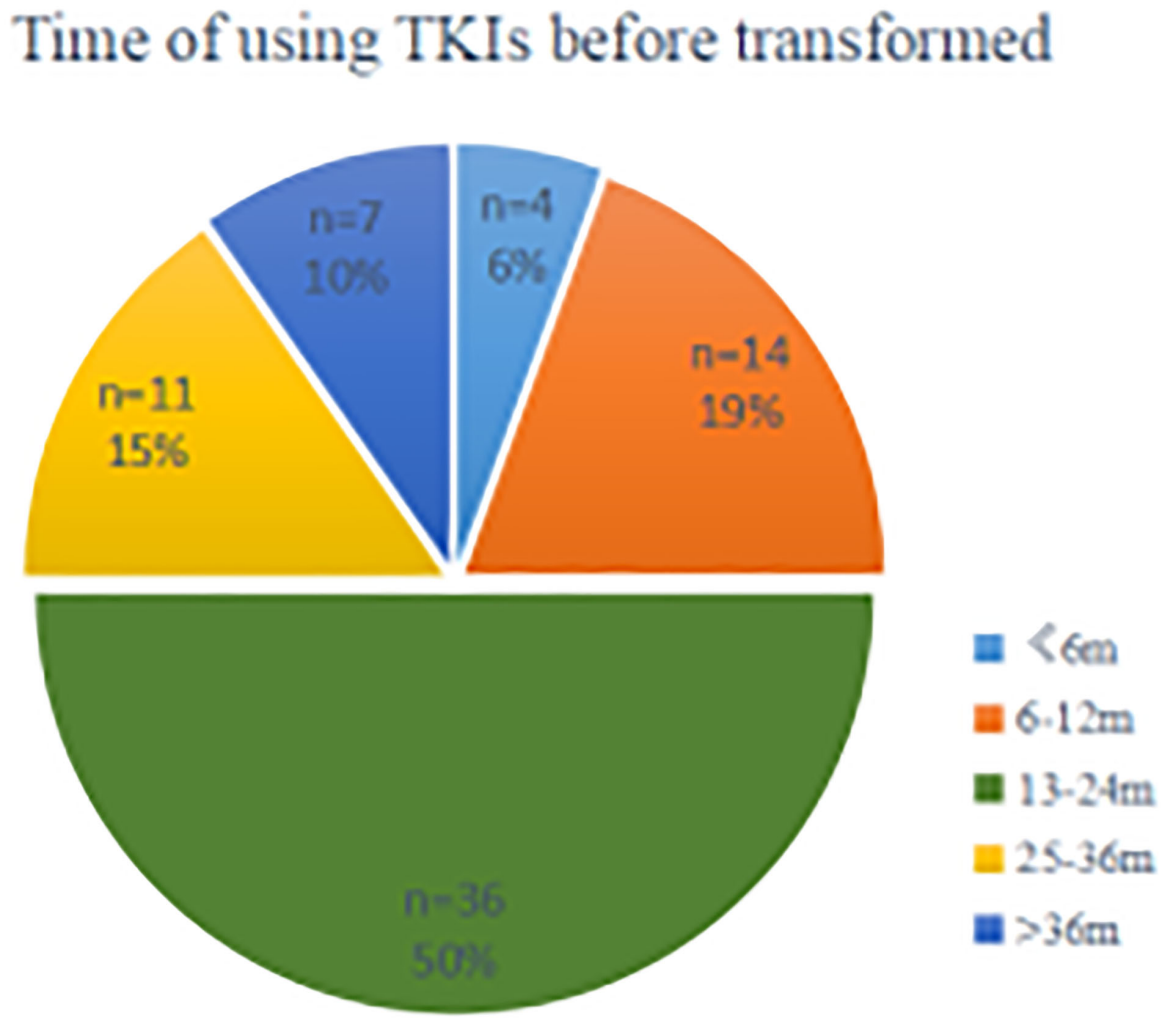
A segmented time scale diagram from the beginning of TKIs using the occurrence of pathological transformation. TKIs, Tyrosine kinase inhibitors; n, number; m, months.

The estimated median time to ttSCLC was 20.5 months (95% CI, 15.45 to 26.55 months), and the estimated median time to T-ttSCLC was 18.5 months (95% CI, 13.86 to 23.39 months) (**Figure 2**). The transformation time of SCLC in most cases is between 6 and 36 months, and 50% of them occur within 1 to 2 years (**Figure 3**).

**Figure 4:**
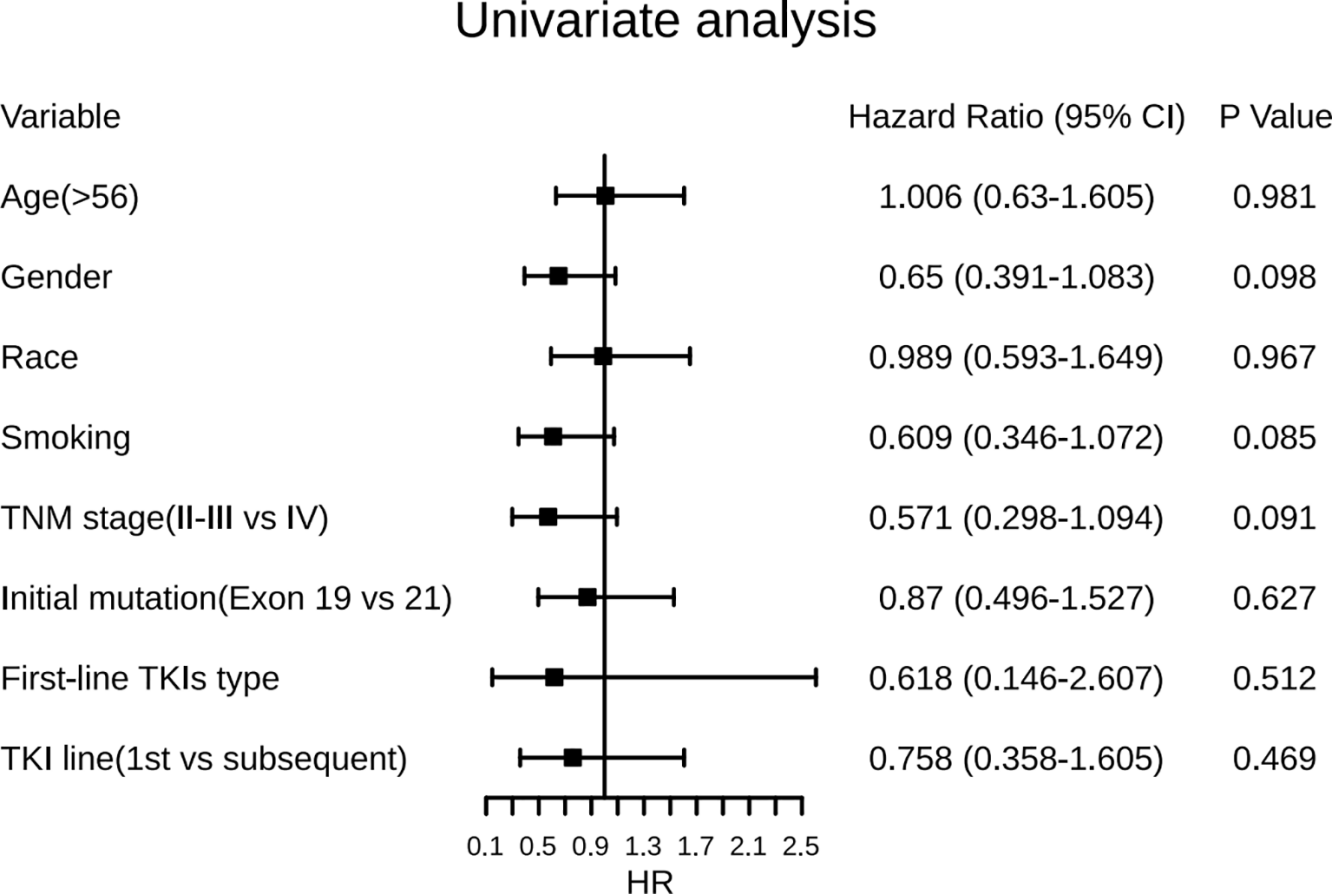
Forest plots of the effect of several factors on time from diagnosis of LADC to development of SCLC phenotype. LADC, Lung adenocarcinoma; SCLC, Small cell lung cancer; TKIs, Tyrosine kinase inhibitors; Exon 19, EGFR exon 19-deletion; Exon 21, EGFR exon 21-L858R.

In the univariate analysis (**Figure 4**), female gender was associated with a predictor of longer ttSCLC of borderline significance (HR, 0.65; 95% CI, 0.391–1.083; *p* = 0.098), while tobacco smoking (either former or current) was also associated with an earlier occurrence of ttSCLC phenotype of borderline significance (HR, 0.61; 95% CI, 0.346–1.072; *p* = 0.085). The initial diagnosis of stage IV cases may have a longer transformation time of SCLC than early and medium-term cases (HR, 0.57; 95% CI, 0.298–1.094; *p* = 0.091). Furthermore, age, race, first-line TKIs type, and initial TKIs line had no significant correlation with ttSCLC and T-ttSCLC.

#### Pre-Transformation Curative Effect

**Figure 5:**
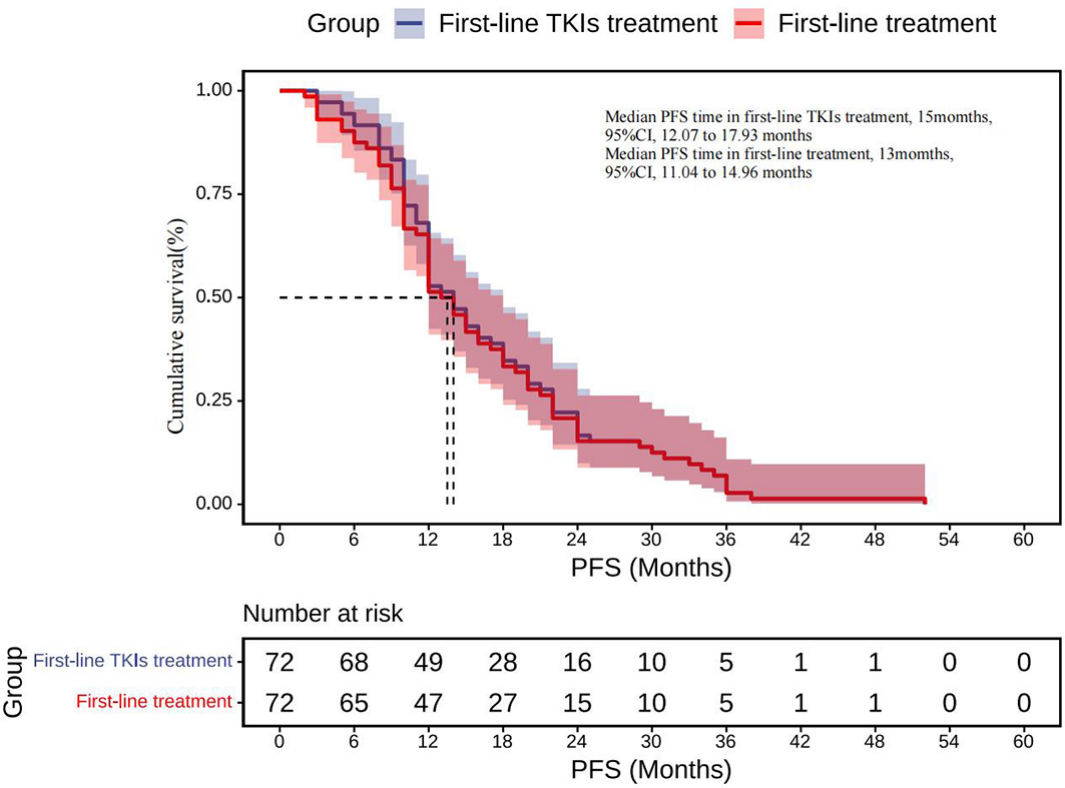
First-line TKIs treatment: Kaplan–Meier estimates of PFS for TKIs first-line therapy; First-line treatment: Kaplan–Meier estimates of total PFS for first-line therapy. SCLC, Small cell lung cancer; TKIs, Tyrosine kinase inhibitors; PFS, Progression-free survival.

The therapy before transformation includes targeted therapy (1st-/2nd-/3rd-generation TKIs), chemotherapy, radiotherapy, and combined treatment. All cases have a history of TKIs therapy and have response efficacy before transformation, and TKIs used in first-line treatment account for 90.3% (**Appendix Table**). The median PFS for TKIs first-line therapy was 15 months (95% CI, 12.07 to 17.93 months), while the total median PFS for first-line therapy was 13 months (95% CI, 11.04 to 14.96months) (**Figure 5**).

### Post-Transformed Characteristics

#### First-Line Treatment and Efficacy

**Figure 6:**
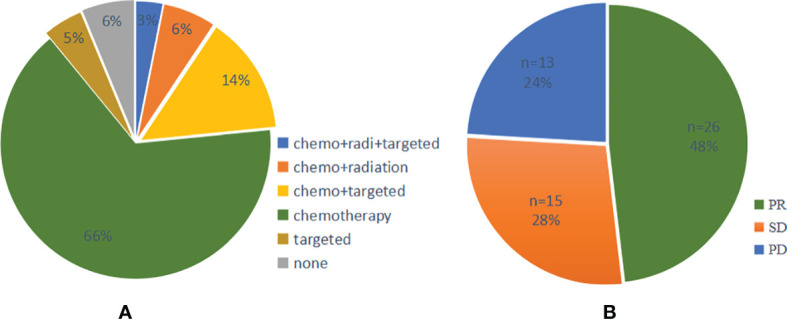
**(A)** Pie chart of first-line treatment and **(B)** efficacy after SCLC transformation. PR, Partial response; SD, Stable disease; PD, Progressive disease; SCLC, Small cell lung cancer.

**Figure 7:**
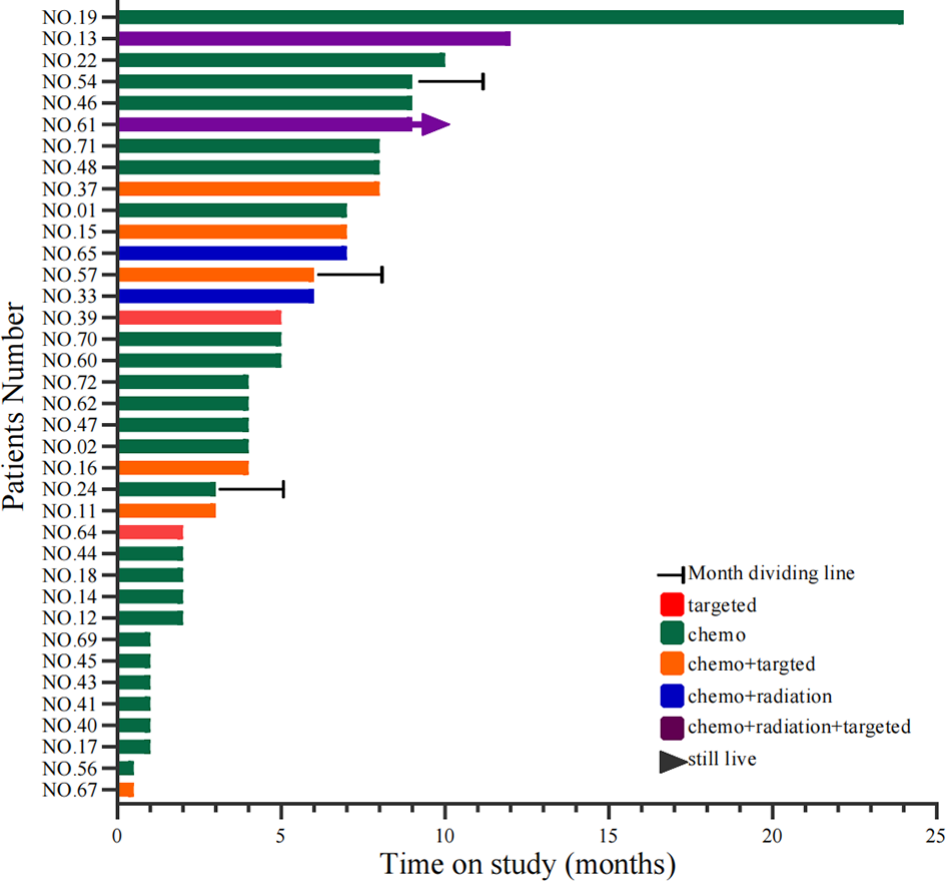
Swimming lane diagram of PFS in first-line treatment after SCLC transformation. PFS, Progression-free survival; SCLC, Small cell lung cancer. Target type: No. 39, osimertinib; No. 64, afatinib; No. 11/15/37/67, erlotinib; No. 16, osimertinib; No. 57, gefitinib.

A total of 64 cases had first-line therapy data after transformation (**Table 2**), of which 42 cases (66%) were classic chemotherapy regimens containing platinum, 9 cases received chemotherapy combined with targeted therapy, 4 cases received chemotherapy combined with radiotherapy, 3 cases continued single-targeted therapy, 2 cases received chemotherapy combined with radiotherapy and targeted therapy, and 4 cases did not receive further treatment. The disease control rate (DCR) of first-line therapy after transformation was 76%, while the objective response rate (ORR) was 48% (**Figure 6**). Thirty-seven patients had specific first-line progression-free survival (PFS) after transformation (**Figure 7**), of which 15 cases have PFS for no more than 3 months, 10 cases for 3 to 6 months, and 12 cases for more than 6 months, including 2 cases for more than 1 year. The median PFS was 4.0 months (95% CI, 3.16 to 7.50 months). However, the median PFS of the combined treatment group was over 6 months. Compared with conventional chemotherapy, combination therapy (including chemotherapy combined with radiotherapy, chemotherapy combined with targeted therapy, and chemotherapy combined with chemotherapy and targeted therapy) has a longer median PFS time (4 months vs. 7 months).

**Table 2:** First-line treatment and efficacy.

First line therapy after transformation	Cases	Median PFS (range)	Ratio of effective cases (ORR)
Chemo+radi+targeted	2	11.5 m (9–12 m)	50%
Chemo+radiation	4	6.5 m (6–7 m)	33%
Chemo+targeted	9	6 m (0.5–8 m)	33%
Chemotherapy	42	4 m (0.5–24 m)	53%
Targeted	3	3.5 m (2–5 m)	50%
None	4	–	–
Total	64	4.0 m (95% CI, 3.16–7.50 m)	48%

Chemo, Chemotherapy; Radi, Radiation; m, months; PFS, Progression-free survival; ORR, Objective response rate.

#### Survival After SCLC Transformation

**Figure 8:**
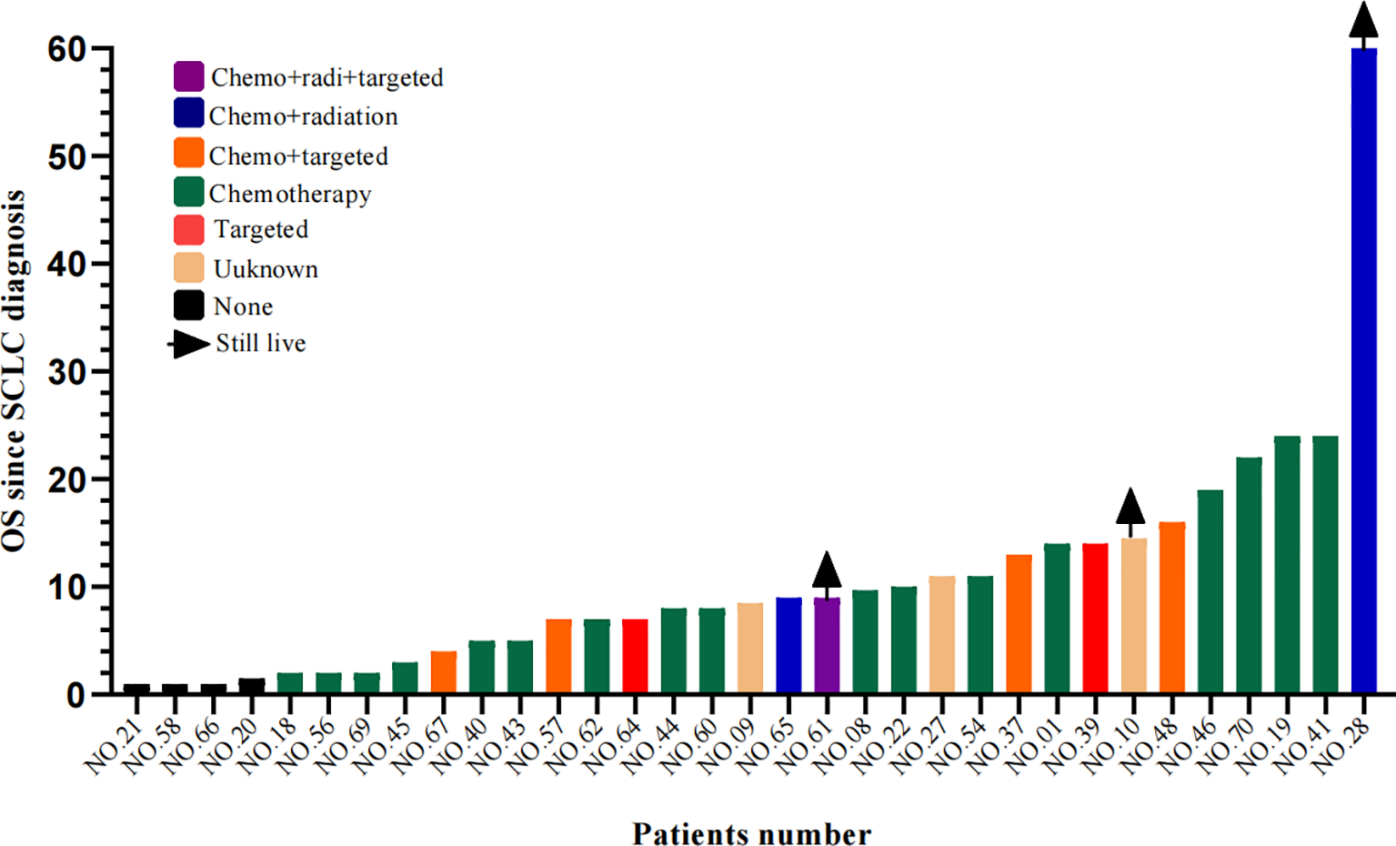
Swimming lane diagram of OS in different therapies after SCLC transformation. OS, Overall survival.

**Figure 9:**
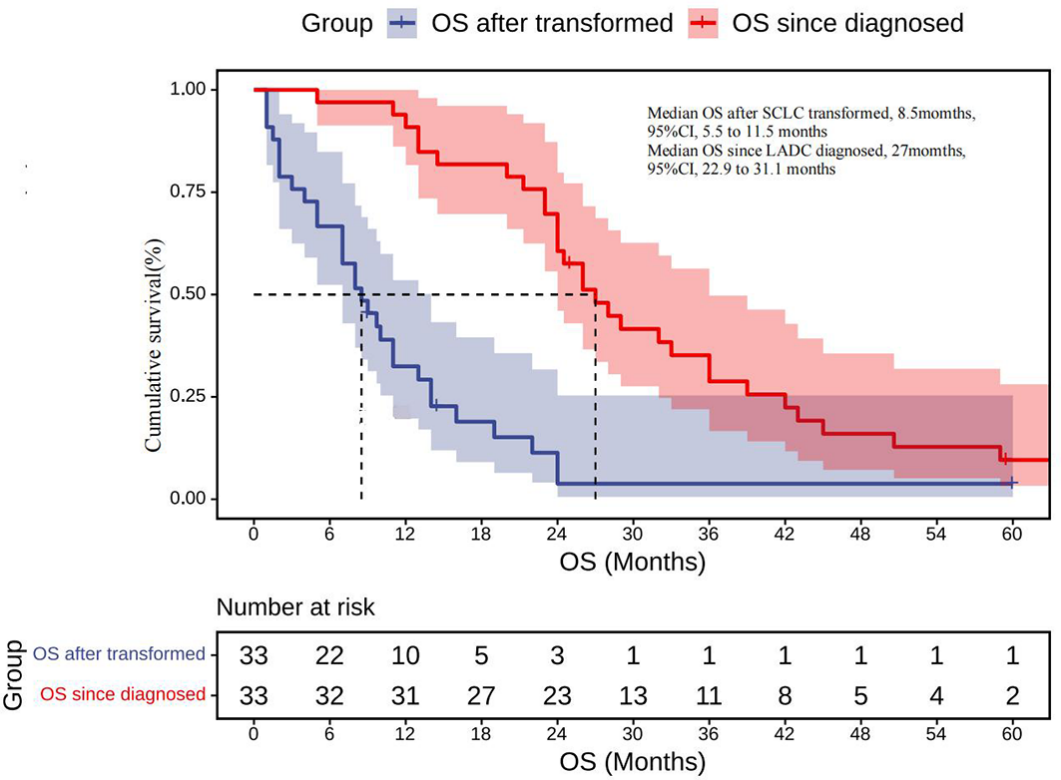
OS after being transformed: Kaplan–Meier estimates of OS since the time of SCLC transformation; OS since diagnosed: Kaplan–Meier estimates of OS since the time of diagnosis. OS, overall survival.

**Figure 10:**
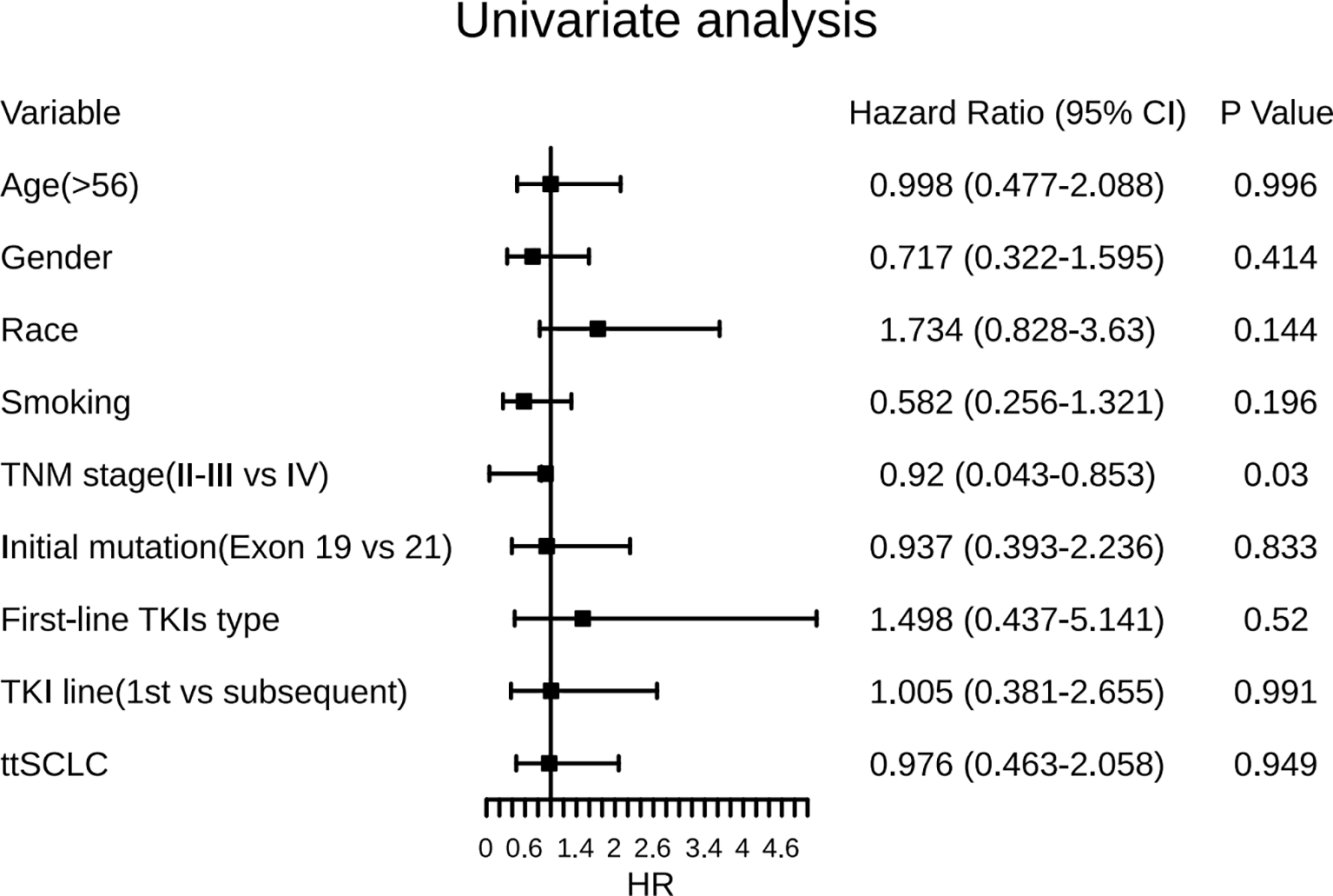
Forest plots of the effect of several factors on OS after development of SCLC phenotype. ttSCLC, the time to small cell lung cancer transformation; TKIs, Tyrosine kinase inhibitors; Exon 19, EGFR Exon 19-deletion; Exon 21, EGFR Exon 21-L858R; OS, overall survival.

Information on survival status after SCLC diagnosis was available in 33 patients (45.8%) (**Figure 8**). The calculated median OS after SCLC diagnosis was 8.5 months (95% CI, 5.50 to 11.50 months) (**Figure 9**). Unlike the median PFS of first-line treatment after transformation, there was no significant difference in the median OS between the conventional chemotherapy group and the combined treatment group (8 months vs. 9 months). The median OS since diagnosis of LADC was 27 months (95% CI, 22.90 to 31.10 months) (**Figure 9**). The initial TNM stage was medium-term, which was the only factor significantly associated with shorter OS after diagnosis of SCLC (HR, 0.92; 95% CI, 0.043–0.853; *p* = 0.030) (**Figure 10**). Gender, age, race, smoking status, initial mutation type, and line of TKIs therapy did not predict the patients’ outcome. Moreover, the ttSCLC (dichotomized at the median value) failed to impact patient survival from the time of SCLC transformation.

## Discussion

Transformation to SCLC is a resistance mechanism to EGFR-TKIs that develops in LADC. A systematic analysis on the transformation of SCLC was published in 2017 (21), including 39 patients. Since then, the literature on this aspect were only case reports or small series of retrospective analyses. There is a lack of research with a large sample size. We systematically searched and summarized the relevant English publications on the transformation of SCLC after EGFR-TKIs resistance in LADC since 2006.

There are generally two hypotheses about the transformation of LADC into SCLC. One hypothesis for the SCT phenomenon is that there is a small amount of SCLC component in LADC patients when they are initially diagnosed. The SCLC component will become dominant if the LADC component was killed by the EGFR-TKIs (4). This is a pseudo-SCT; although this hypothesis existed for a long time, strong evidence is still lacking. On the contrary, the second hypothesis, that both LADC and SCLC may arise from identical cell clones, is widely accepted (16). Previous studies have found that most transformed SCLC samples have their original EGFR mutation (4). It was reported that targeted disruption of TP53 and RB1 in alveolar type II cells led to the development of SCLC (62). In our study, 67 cases had gene detection results after SCLC transformation, of which 87% retained EGFR 19-del or 21-L858R mutation. However, it is noteworthy that 17 cases had combined T790M mutation before transformation, but only 1 case retained T790M mutation after transformation (Appendix A). The relative absence of T790M is consistent with the loss of EGFR dependence in transformed SCLC, as reported by Offin (63), and whether the loss of T790M mutation is related to SCLC transformation needs further study.

To determine that TKIs resistance is caused by SCLC transformation, we formulated a stricter inclusion criteria than that in the 2017 article (4), which requires each case to receive TKIs treatment for at least 3 months. The efficacy evaluation is at least SD. We excluded cases where the initial pathology contained components of small cell lung cancer. A total of 72 cases were included in our study. To our knowledge, this cohort represents the largest report to date of clinical outcomes for patients with EGFR-sensitive mutations LADC who developed SCLC transformation after TKIs resistance.

We found that the baseline demographic characteristics of people who have undergone SCLC transformation are similar to those of people who have not undergone transformation, which is different from the findings of previous studies (4, 64). As mentioned above, apart from the T790M mutation, most transformed SCLC cases maintained the same EGFR mutation present in the former LADC, including 18-G719X, 19-deletion, and 21-L858R mutations. EGFR mutations are extremely rare in primary SCLC (65). The persistence of the same EGFR mutation suggests that the SCLC phenotype is clonally derived from the primary LADC, providing further evidence that the transformed SCLC component originated from the LADC component, not the original one.

We observed that SCLC transformation occurs over a wide time span, seen as early as 3 months and as late as more than 6 years after the diagnosis of LADC. However, the median time to transformation was 20.5 months. Seventy-five percent of cases received TKIs therapy for more than 12 months before SCLC transformation. The median PFS of first-line TKIs treatment was 14 months, which appears to be longer than the PFS reported in patients treated with TKIs in prospective clinical trials (8–11 months). This observation suggests that patients obtaining a long-term benefit with these drugs are at a higher risk of trans-differentiating to an SCLC phenotype at progression. We also analyzed factors potentially predicting SCLC transformation; however, no significant factors were found to be associated with SCLC transformation. Differently from previous studies, our study shows that the female gender is only borderline related to a longer ttSCLC, while smoking status is also borderline related to the trend of early SCLC phenotype when progressing to TKIs treatment, while other factors are not significantly related to the length of ttSCLC.

After transformation, clinical behavior mimics classic (EGFR wild-type) SCLC on many levels, with frequent but transient responses to platinum etoposide. The median PFS was 4 months after first-line treatment and the median OS was 8.5 months. Compared with primary SCLC, the clinical prognosis of transformed SCLC is worse (66). We found that combination therapy (including chemotherapy combined with radiotherapy, chemotherapy combined with targeted therapy, and chemotherapy combined with chemotherapy and targeted therapy) had a longer median PFS than conventional chemotherapy (4 months vs. 7 months). However, there was no significant benefit in the combined treatment group in the transformed median OS.

The overall prognosis of transformed SCLC is poor, so it is urgent to explore more treatment methods. Currently, immunocombination therapy has become a new standard for the first-line treatment of extensive SCLC with the median OS reaching 15 months (67). Whether transformed SCLC can also benefit from immune checkpoint inhibitors needs further clinical exploration and research.

The limitations of our study must be noted, i.e., that the patient data were retrospectively collected from published articles. The retrospective nature of this study may have influenced some results, such as treatments and response assessments. Due to the limitations of this retrospective study, some cases lack the data to be analyzed, and it is impossible to obtain samples for further detection and analysis. Our center has been conducting a prospective case inclusion study and will further explore the differences in the expression of the whole gene spectrum before and after SCLC transformation to find the relevant driver genes of SCLC transformation.

## Conclusion

Our pooled analysis shows that the realization of SCLC phenotype mostly takes more than 1 year, and the prognosis of patients after SCLC diagnosis is worse than primary SCLC. Chemotherapy combined with targeting and radiotherapy can improve PFS of transformed SCLC but cannot benefit OS. In the future, novel therapies must be explored.

## Data Availability Statement

The original contributions presented in the study are included in the article/**Supplementary Material**. further inquiries can be directed to the corresponding author.

## Author Contributions

Concept and design: JX and LX. Article writing: JX. Extraction and collection of data: JX and LX. Statistical analysis: JX and LX. Drafting and revision of manuscript: JX and ZY. Supervision: ZY. Final approval of the manuscript: JX, XL, BW, WK, YC, and ZY. All authors contributed to the article and approved the submitted version.

## Funding

This work was supported by grants from the Clinical Key Specialty Construction Project of Fujian Province (No. 2015-593 and No. 2017YZ0001-2) and the 900th Hospital of the Joint Logistic Support Force of China: International Cooperative Research Program (Grant/Award Number: 2017L03).

## Conflict of Interest

The authors declare that the research was conducted in the absence of any commercial or financial relationships that could be construed as a potential conflict of interest.

## Publisher’s Note

All claims expressed in this article are solely those of the authors and do not necessarily represent those of their affiliated organizations, or those of the publisher, the editors and the reviewers. Any product that may be evaluated in this article, or claim that may be made by its manufacturer, is not guaranteed or endorsed by the publisher.
